# Host chemokine signature as a biomarker for the detection of pre-cancerous cervical lesions

**DOI:** 10.18632/oncotarget.24946

**Published:** 2018-04-06

**Authors:** Ramya Bhatia, Kim Kavanagh, June Stewart, Sharon Moncur, Itziar Serrano, Duanduan Cong, Heather A. Cubie, Juergen G. Haas, Camille Busby-Earle, Alistair R.W. Williams, Sarah E.M. Howie, Kate Cuschieri

**Affiliations:** ^1^ Human Papillomavirus Research Group, Division of Pathology, University of Edinburgh, Edinburgh, United Kingdom; ^2^ Department of Mathematics and Statistics, Strathclyde University, Glasgow, United Kingdom; ^3^ Centre for Inflammation research, University of Edinburgh, Edinburgh, United Kingdom; ^4^ Division of Infection and Pathway Medicine, University of Edinburgh, Edinburgh, United Kingdom; ^5^ Global Health Academy, University of Edinburgh, Edinburgh, United Kingdom; ^6^ Simpson Centre for Reproductive Health, Royal Infirmary of Edinburgh, Edinburgh, United Kingdom; ^7^ Scottish HPV Reference Laboratory, NHS Lothian, Royal Infirmary of Edinburgh, Edinburgh, United Kingdom

**Keywords:** chemokines, HPV, cervical screening, triage, biomarkers

## Abstract

**Background:**

The ability to distinguish which hrHPV infections predispose to significant disease is ever more pressing as a result of the increasing move to hrHPV testing for primary cervical screening. A risk-stratifier or “triage” of infection should ideally be objective and suitable for automation given the scale of screening.

**Results:**

CCL2, CCL3, CCL4, CXCL1, CXCL8 and CXCL12 emerged as the strongest, candidate biomarkers to detect underlying disease [cervical intraepithelial neoplasia grade 2 or worse (CIN2+)]. For CIN2+, CCL2 had the highest area under the curve (AUC) of 0.722 with a specificity of 82%. A combined biomarker panel of six chemokines CCL2, CCL3, CCL4, CXCL1, CXCL8, and CXCL12 provides a sensitivity of 71% and specificity of 67%.

**Conclusion:**

The present work demonstrates that the levels of five chemokine-proteins are indicative of underlying disease. We demonstrate technical feasibility and promising clinical performance of a chemokine-based biomarker panel, equivalent to that of other triage options. Further assessment in longitudinal series is now warranted.

**Methods:**

A panel of 31 chemokines were investigated for expression in routinely taken archived and prospective cervical liquid based cytology (LBC) samples using Human Chemokine Proteomic Array kit. Nine chemokines were further validated using Procartaplex assay on the Luminex platform.

## INTRODUCTION

Persistent infection with high-risk (oncogenic) Human Papilloma Virus (hrHPV) causes >95% of cervical cancers [1]. Cervical hrHPV infection is common but most infections clear naturally. In a small proportion of women, however, infection persists and persistent infection can lead to the development of cervical lesions referred to as cervical intraepithelial neoplasia (CIN grades 1 to 3 according to increasing severity) [2, 3]. If left untreated a proportion of lesions will develop into cervical cancer. Around thirteen hrHPV types cause 99% of all cervical cancers. Two types, HPV16 and HPV18 cause over 70% of cancers [4].

In women who fail to clear infection it is reasonable to hypothesise that the local immune response profile may differ from that in women who have cleared infection. In particular, innate immune response proteins such as chemokines, produced by cervical cells should be detectable in proteins extracted from cervical liquid based cytology (LBC) samples. Chemokines are produced in response to any hrHPV infection independent of underlying type (unlike antibodies or T cell responses) and are made in high amounts during persistent viral infection [5]. Their detection may therefore serve as a biomarker to distinguish clinically significant infection and associated disease.

Cervical screening is changing globally from a cytology based approach to the molecular detection of hrHPV types [6–9]. While the sensitivity of hrHPV testing for CIN2+ has been demonstrated, the specificity of hrHPV testing to detect disease is sub-optimal. Several triage tests have been suggested to risk-stratify women who are hrHPV positive, including cytology and HPV 16/18 genotyping (where women with 16/18 infection are sent to colposcopy and women with other high risk are managed conservatively) [10]. However, there is international consensus that better triage tests are needed urgently to determine which hrHPV positive women are truly at risk of significant disease [11].

The aim of the present work was to determine whether chemokines are differentially expressed in women with and without cervical disease in order to determine the feasibility of this approach for the risk-stratification of hrHPV infection.

## RESULTS

### Chemokines are differentially expressed in different grades of hrHPV induced CIN

**Figure 1:**
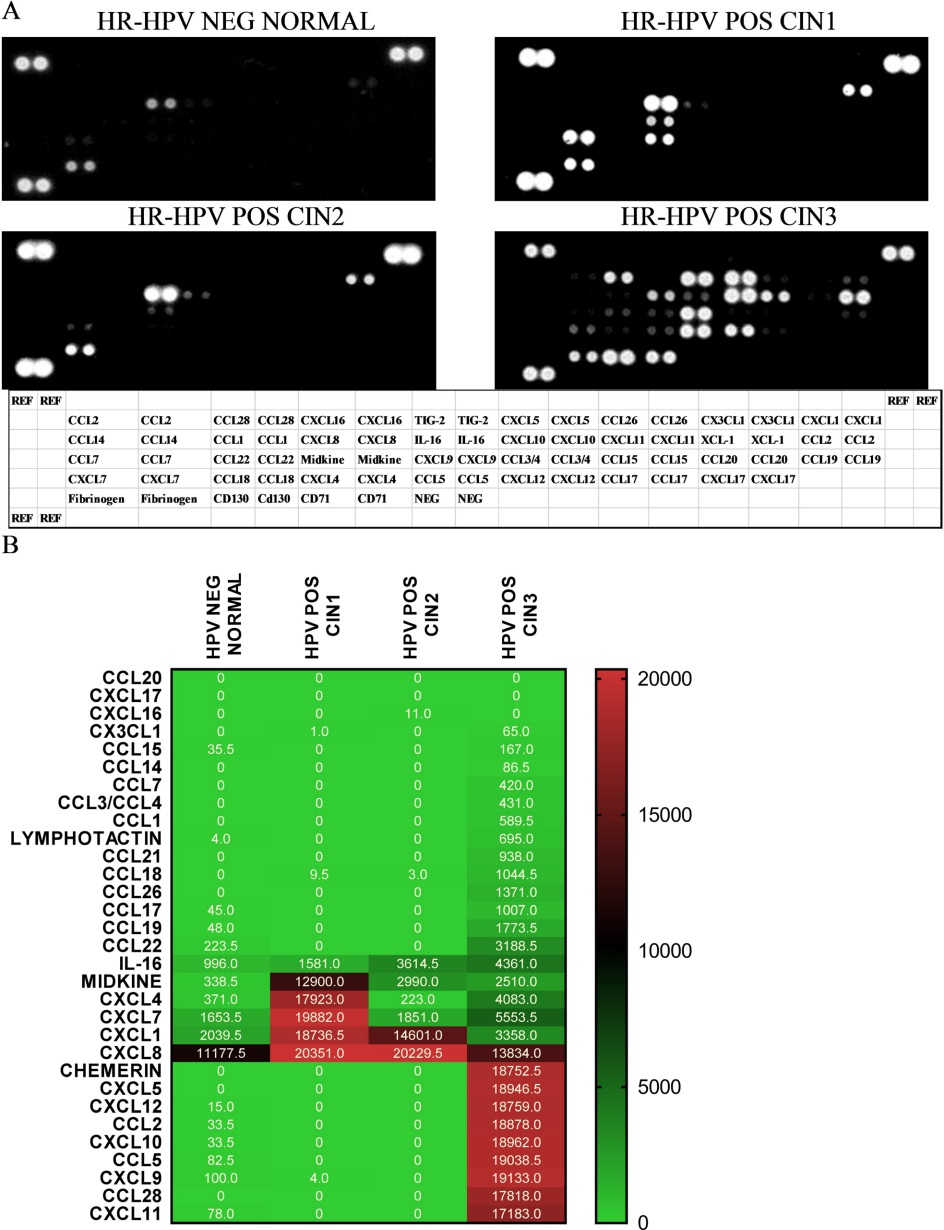
Proteomic array shows detection of chemokines in pooled aliquots from 9 samples in following categories hrHPV negative, cytological normal; hrHPV positive, CIN1; 3) hrHPV positive CIN2; and 4) hrHPV positive CIN3 (**A**). Relative pixel density ratio for each protein on the array was calculated using ImageJ by normalising to negative controls (NEG CONTROL) (**B**). Heatmap representing array results with pixel density.

A proteomic array including 31 chemokines was tested on pooled protein extracts from LBC samples associated with different CIN grades. (Figure 1). Out of 31 chemokines on the array, CCL2, CCL5, CCL28, CXCL5, CXCL9, CXCL10, CXCL11, CXCL12 and Chemerin were upregulated in the samples from women with CIN3 compared to samples associated with negative HPV and normal cytology status. A total of three chemokines (Midkine, CXCL4, CXCL7) were highly expressed in CIN1 but not in CIN2 or CIN3; and CXCL1 was highly expressed in CIN1 and CIN2 but not in CIN3. CXCL8 was highly expressed in all sample categories. Based on these results, a combination of chemokines was selected for further analysis.

### Chemokine expression levels are indicative of disease severity

**Figure 2:**
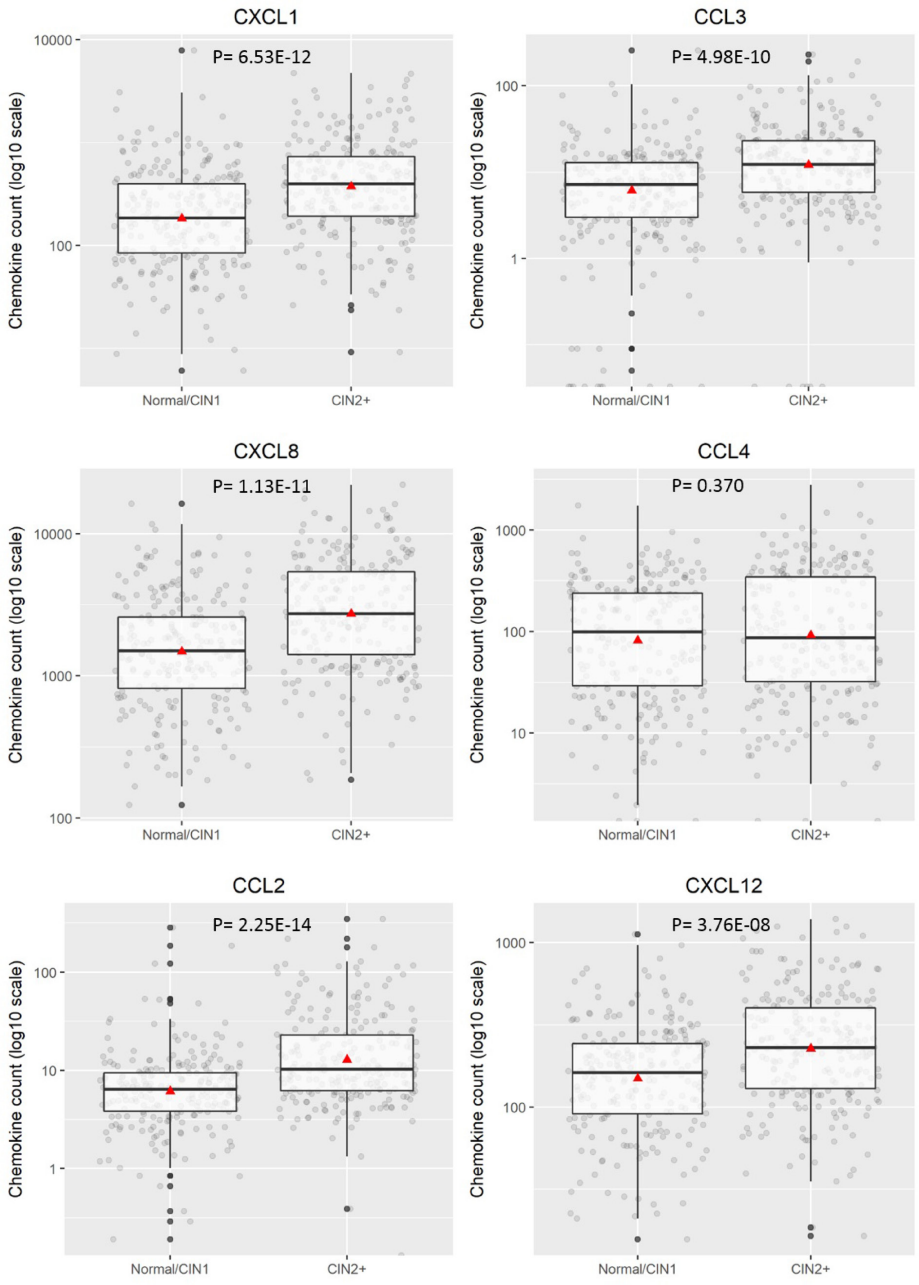
Distribution of chemokine level (pg/200 ug) for ≤CIN1 versus CIN2+ Boxplots demonstrate the median, lower and upper quartile of the values, with the mean value represented as a triangle. Individual data points are shown as grey dots. Values deemed to be outliers of the distribution are shown as black dots. *P* values were calculated using Mann–Whitney test.

**Figure 3:**
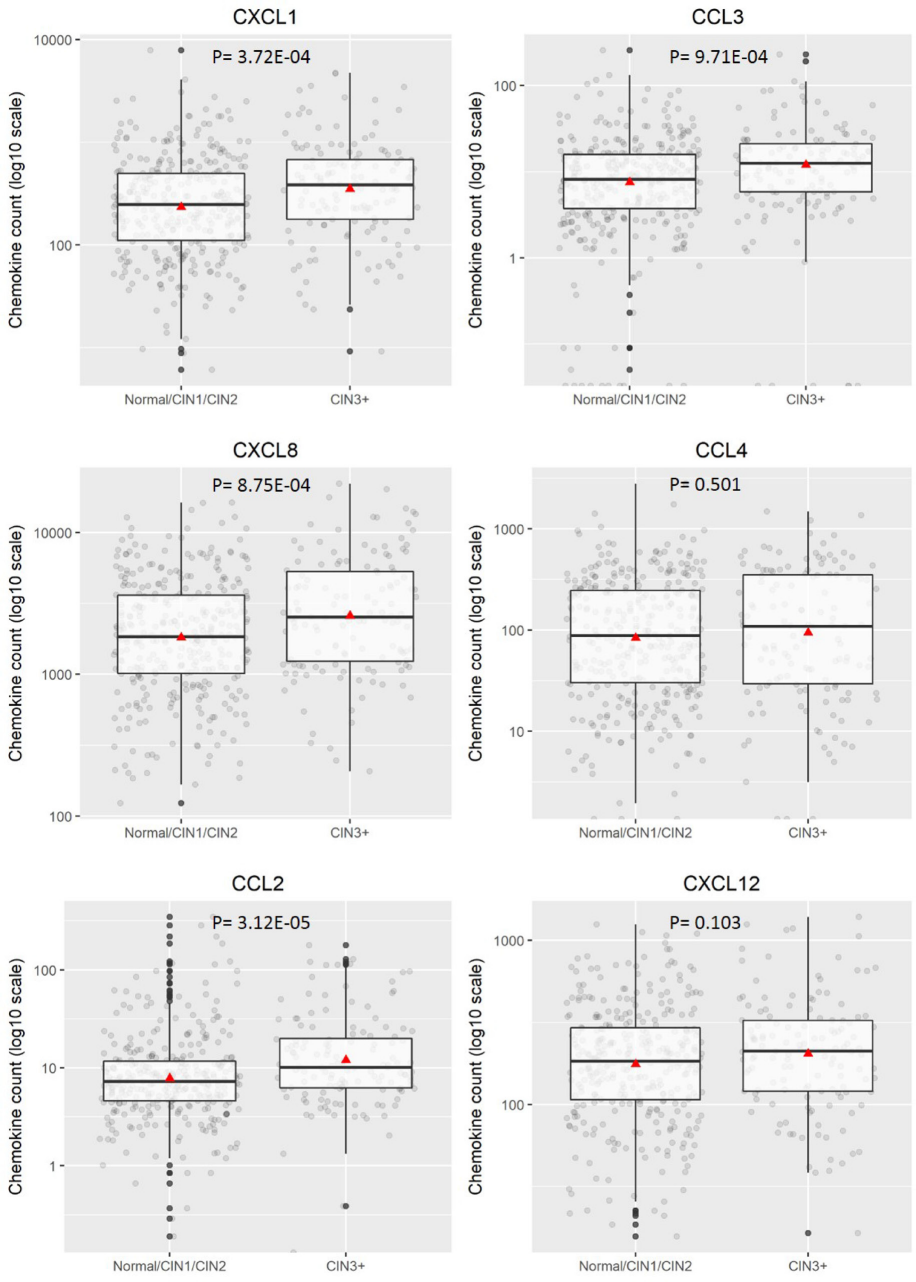
Distribution of chemokine level (pg/200 ug) for categorised disease groupings hrHPV positive ≤CIN2 versus CIN3+ Boxplots demonstrate the median, lower and upper quartile of the values, with the mean value represented as a triangle. Individual data points are shown as grey dots. Values deemed to be outliers of the distribution are shown as black dots. *P* values were calculated using Mann–Whitney test.

To investigate quantitative expression of chemokines in LBC samples and to validate results from the proteomic array, a multiplex bead-based Procartaplex™ assay was performed. As indicated in the Methods section, the commercially available 8-plex Procartaplex assay used for validation included five chemokines of interest identified in the proteomic array - CCL2, CXCL1, CXCL8, CXCL10 and CXCL12 and also three others – CCL3, CCL4, CCL11. CCL11 and CXCL10 were below the threshold for measurement by the multiplex assay and CCL4 expression levels showed no significant difference between any disease categories (*p* = 0.37 for CIN2+ and *p* = 0.501 for CIN3+). However, CCL2, CCL3, CXCL1, CXCL8 and CXCL12 all showed significantly different expression with higher levels of expression generally observed in CIN2+ (*p* < 0.0001, Figure 2). When assessing CIN3+ as outcome (compared to ≤CIN2. Figure 3), CCL2, CCL3, CXCL1 and CXCL8 showed significantly higher expression (*p* < 0.0001) whereas CXCL12 (*p* = 0.061) did not.

### Individual and combined clinical performance of chemokines for the detection of CIN2+ and CIN3+

Univariate analysis was performed for each chemokine individually to identify the sensitivity and specificity for the detection of CIN2+ and CIN3+ (Table 1A). Sensitivity, specificity and overall predictive ability of each chemokine varied dependent on the disease threshold considered. For CIN2+, CCL2 has the highest AUC of 0.722 with sensitivity and specificity of 55.6% and 82% respectively. CXCL1 provided the highest sensitivity (73.4%) and CCL2 offered the highest specificity (82%). For CIN3+ as the disease threshold, CCL2 had the highest AUC of 0.648 with sensitivity and specificity of 59.5% and 64% respectively. CXCL8 had the highest sensitivity (60.8%) and CCL3 had the highest specificity (70.8%).

**Table 1A:** Univariate analysis summarising the individual ability of each chemokine to predict the disease state based on two disease cut-offs –CIN2+ and CIN 3+

	CIN2+^*^				CIN3+^**^			
Chemokine	AUC	Accuracy	Sensitivity	Specificity	AUC	Accuracy	Sensitivity	Specificity
CCL2	0.722	68.1%	55.6%	82.0%	0.648	62.55%	59.46%	63.98%
CCL3	0.699	66.8%	56.5%	78.4%	0.647	66.38%	56.76%	70.81%
CCL4	0.469	47.7%	38.7%	57.7%	0.528	63.83%	32.43%	78.26%
CXCL1	0.706	67.2%	73.4%	60.4%	0.645	65.96%	59.46%	68.94%
CXCL8	0.660	63.8%	52.4%	76.6%	0.595	57.02%	60.81%	55.28%
CXCL12	0.646	62.6%	58.1%	67.6%	0.566	48.09%	81.08%	32.92%

^*^Proportion of CIN2+ in test data set, 53%. ^**^Proportion of CIN3+ in test data set 31.5%.

**Table 1B:** Summary of model predictability where combinations of chemokines are considered in a logistic regression framework

	CIN2+^*^				CIN3+^**^			
Model	AUC	Accuracy	Sensitivity	Specificity	AUC	Accuracy	Sensitivity	Specificity
Model 1: All 6 chemokines	0.704	66.81%	67.74%	65.77%	0.580	62.13%	48.65%	68.32%
Model 2: All 6 chemokines plus interactions CXCL8*CXCL1 and CCL3*CCL4	0.726	68.51%	59.68%	78.38%	0.494	31.91%	83.78%	8.07%
Model 3: All 6 chemokines plus interaction CXCL8*CXCL1	0.711	66.38%	54.84%	79.28%	0.522	63.40%	35.14%	76.40%
Model 4: All 6 chemokines plus interaction CCL3*CCL4	0.723	68.94%	70.97%	66.67%	0.546	61.28%	48.65%	67.08%
Model 5: CXCL8 and CCL2	0.696	65.96%	58.87%	73.87%	0.601	58.72%	62.16%	57.14%
Model 6: CXCL1 and CCL2	0.752	69.36%	57.26%	82.88%	0.667	65.53%	63.51%	66.46%
Model 7: CXCL1 and CCL2 and interaction CXCL1*CCL2	0.752	69.79%	58.06%	82.88%	0.669	65.11%	66.22%	64.60%
Model 8: CXCL1 and CXCL8	0.687	64.68%	49.19%	81.98%	0.618	57.45%	72.97%	50.31%
Model 9: CXCL1 and CXCL8 and interaction CXCL1*CXCL8	0.703	65.53%	49.19%	83.78%	0.561	58.72%	50.00%	62.73%
Model 10: CXCL1, CXCL8 and CCL2	0.709	66.38%	52.42%	81.98%	0.619	58.30%	72.97%	51.55%

*Proportion of CIN2+ in test data set, 53%. ^**^Proportion of CIN3+ in test data set 31.5%.

A multivariate model using a logistic regression framework was designed to identify potential chemokine biomarker combinations which were predictive of disease. Of the interactions considered, two significant interactions were found using the CIN2+ disease threshold; CXCL1* CXCL8 (*p* = 0.000135) and CCL3* CCL4 (*p* = 0.000119). No significant interactions were found for the CIN3+ thresholds but for purposes of comparability, these interactions were also considered for the CIN3+ outcome (Table 1B). Using a model of all 6 chemokines plus interaction between CCL3 and CCL4 (model 4) provided the best predictability with a sensitivity and specificity of 70.97% and 66.67% (for CIN2+) and 48.65% and 67.08% (for CIN3+) respectively. Models 6 and 7 have a high AUC (75% for CIN2+ and 67% for CIN3+). Using the two biomarkers CCL2 and CXCL1 provides high specificity of 82% (for CIN2+) and 66% (for CIN3+) but with a lower sensitivity.

### Clinical performance of HPV16/18 genotyping for the detection of CIN2+ and CIN3+

For the 483 hrHPV positive women in our sample set, we had HPV16/18 limited genotyping information using the Abbott RealTime HPV test. According to this, 155/239 CIN2+ and 99/ 138 CIN3+ had a 16/18 infection. Sensitivity and specificity of HPV16/18 genotyping was 64.8% (95% CI: 58.4–70.8) and 43.4% (95% CI: 37.1–49.9%) for CIN2+ and 71.7% (95% CI: 63.6–78.9) and 43.4% (95% CI: 38.4–49.1%) respectively for CIN3+.

## DISCUSSION

Currently the most evidenced triage options for hrHPV positive women detected at screening are cytology with or without adjunctive p16/Ki67 staining, HPV16/18 genotyping and viral and host DNA methylation markers [12]. However, none of the above satisfy all the requirements of an ideal triage test: - 1) high specificity for CIN2+; 2) objectivity; 3) high-throughput suitable for automation; 4) amenable to a wide range of biospecimen types and 5) produced independently of the infecting virus type. Therefore, there is urgent need for new biomarkers to risk-stratify hrHPV positive women.

The immune response plays a central role in clearing transient HPV infections. Persistent infection and progression to cervical cancer occurs in only a minority of hrHPV positive women. Elevated levels of pro-inflammatory chemokines are indicative of persistent HPV infection and associated carcinogenesis [13]. Several of these pro-inflammatory chemokines such as CXCL1, CXCL8 and CXCL12 drive cancer progression by facilitating cell growth, survival and migration and inducing angiogenesis [14–17]. Other chemokines such as CCL2, CCL3, CCL4 promote tumour invasion and metastases [18–23].

Our results show that chemokine signature differs with severity of disease. Certain chemokines CCL2, CCL5, CCL28, CXCL5, CXCL9, CXCL10, CXCL11, CXCL12 and Chemerin were upregulated in the samples from women with CIN3 vs no disease. On the other hand, chemokines Midkine, CXCL4, CXCL7 were highly expressed in CIN1 but not in CIN2 or CIN3 and CXCL1 was highly expressed in CIN1 and CIN2 but not in CIN3. CXCL8 expression also increased with severity of disease. Validation of these findings was performed on a multiplex Bioplex assay which showed that CCL2, CCL3, CXCL1, CXCL8 and CXCL12 were upregulated in hrHPV positive women with CIN2+ compared to hrHPV positive women with negative cytology or CIN1 histology. Our study backs previous findings by Cicchini *et al* [24] who showed, using gene expression data from human cervical tissue specimens, that CCL3, CCL19, CXCL8, CXCL9 and CXCL11 mRNA levels increased progressively with disease severity while CCL20, CXCL1, CXCL2, CXCL5, and CXCL6 were upregulated in early transition from normal to CIN1/2 and CCL8 and CXCL12 increased in transition to invasive tumours. Further, expression of CXCL1, CXCL2, CXCL8, CXCL10, and CXCL11 was significantly increased in W12G and W12GPXY cells compared to NIKS [24]. While both W12 and NIKS cells are transformed human keratinocyte cell lines, W12 cell lines have HPV-16 while NIKS do not. This indicates a role of HPV in the overexpression of these chemokines. Other small scale studies by Zanotta *et al* shows that CCL7, CXCL9, CXCL12 were overexpressed in women with cytological abnormalities [23] and Iwata *et al* shows that CXCL8 was upregulated in High grade squamous intraepithelial neoplasia (HSIL) compared to Low grade squamous intraepithelial neoplasia (LSIL) [25].

In the era of HPV primary screening, there is a need for robust triage systems which have a high specificity for disease. Our univariate analysis indicates that CCL2 and CXCL8 can act as individual biomarkers with a specificity for detection of CIN2+ of over 75%. Multivariate analysis using combined biomarkers show that Model 4 measuring all 6 chemokines plus an interaction term between CCL3 and CCL4 gives a sensitivity of 70.97% and specificity of 66.67% for detection of CIN2+. Within our sample set, we compared performance of the chemokine panel with HPV16/18 limited genotyping as a triage strategy. For CIN2+, the sensitivity of the combined chemokine marker set (70.97%) is higher than the sensitivity of HPV16/18 limited genotyping (64.8%) as is its specificity (66.67% v 43.4%). Similar results were seen for a CIN3+ disease threshold.

A strength of our study is its relatively large sample size and level of disease annotation, compared to other feasibility studies that have measured immune biomarkers of cervical disease [23–30]. Our study also made use of samples of the type routinely used for cervical screening programme in the UK and beyond. One of the challenges of using a quantitative protein-based assay is that a method of standardisation/normalisation is required to account for inter-sample variability. For this purpose, we standardised the amount of protein input per sample. Further, we incorporated a technical validation control through the assessment of CXCL8.

However, the present work has certain limitations which we aim to address in the future. The sample incorporated archived samples, some of which had been stored for over 5 years. This may have contributed to the 10% invalidity rate which in a routine setting would be unacceptable. Additionally, the sample set was enriched for disease given the incorporation of a colposcopy population and was not, therefore, representative of a primary screening context. This said, feasibility assessment of candidate biomarkers in a high-prevalence disease setting has precedent and is consistent with overarching cancer biomarker development road-maps [31] and also road-maps specific to the evaluation of new biomarkers for cervical screening [32].

The data presented demonstrate that certain chemokine proteins are associated with high grade disease and can be measured in routinely taken cervical clinical samples. Further, the pattern of chemokine association reconciles with data from mechanistic studies that have shown a functional role of these proteins in tumorigenic processes. While the chemokine biomarker panel shows promise in its performance for detection of CIN2+ and CIN3+, there is scope for improvement. Future work is focussed on further optimisation of the assay to improve the clinical performance. This will involve looking at alternate antibodies and platforms for detection and improving technical sensitivity of the assay. Another stream of work is ongoing investigation of expression levels of the chemokines at the mRNA level to assess their suitability as a triage test. Our results here show that further investigation of the biomarkers is certainly warranted, including in a primary screening prospective setting to further validate this novel and objective approach to the risk-stratification of HPV infection.

## MATERIALS AND METHODS

### Sample collection

The study involved an initial screening (pilot study) of chemokines using Human Chemokine proteomic array on a small panel of LBC samples (*N* = 36). Following on from this, a validation study was carried out using ProcartaPlex multiplex assay on a larger panel of samples (*N* = 1051).

The target sample size for the validation study was calculated *a priori* to be 1050 samples, of which at least ~30% would be CIN2+ and ~30% normal or CIN1 (on follow-up histological assessment). The sample set was designed to incorporate samples from a prospective colposcopy cohort, where colposcopy had been indicated due to preceding abnormal cytology (*N* = 500) in addition to samples from the Scottish HPV Archive (*N* = 550). This sample size gave the ability to estimate with 95% confidence a test with sensitivity of 0.85 and specificity of 0.75 to within ±5%. The final analysis was comprised of 378 samples from the colposcopy cohort and larger number of archived samples (*N* = 673) to achieve the desired sample size.

All cervical samples were collected into ThinPrep-Preservcyt™ liquid based cytology transport medium (Hologic, Crawley, UK). Samples collected at the colposcopy clinic were aliquoted within 24 hours of collection and stored at −80° C. Archived samples were obtained as 1.5 ml aliquots that had been stored at −80° C for 3–5 years and underlying cytopathology and histopathology information (where indicated) was obtained. Cytology classification was according to British Association for Cytopathology criteria. Cytology results were classed as negative (for any abnormality), low grade (borderline squamous changes, koilocytosis, and low grade dyskaryosis) and high grade (which includes moderate dyskaryosis and worse) [2]. Histology results (both for prospective and archived samples) were obtained and classified as -No biopsy taken, Normal biopsy, CIN1, CIN2, CIN3. No histology result was obtained for women with negative cytological assessment.

### Governance

Anonymised archived liquid based cytology (LBC) cervical smear samples were obtained from the Scottish National HPV Archive (http://www.shine.mvm.ed.ac.uk/archive.shtml), which holds Research Ethics Committee approval for Research Tissue banks (REC Ref 11/AL/0174) for provision of samples for HPV related research after approval from the Archive Steering Committee. Samples were made available for this study through approval (HPV Archive Application #0014). Favourable ethical opinion for the prospective colposcopy sample collection was also obtained (REF 12/SS/0034).

### HPV testing

All samples were analysed for hrHPV infection using the RealTime High Risk HPV Assay (Abbott Molecular, US) following the manufacturer's instructions. This assay is considered clinically validated for use in primary cervical screening [33], detects 14 high risk types and offers separate, concurrent genotyping of HPV types 16 and 18.

### Extraction of protein from LBC Samples

Protein was extracted from all 1051 LBC samples using the chloroform methanol extraction method published previously [34]. Protein concentration was determined by Pierce BCA assay (Thermo Scientific, UK).

### Human chemokine proteomic array

Antibody-based Human Chemokine Proteomic Array kit (Cat. No. ARY017, R & D systems, USA) was used to test expression of chemokines in LBC samples. Samples were categorised into the following groups 1) hrHPV negative, cytology negative; 2) hrHPV positive, CIN1; 3) hrHPV positive CIN2 and 4) hrHPV positive CIN3. Protein extracts from nine archived samples (10 μg each) associated with each category were pooled and tested. The assay was performed as previously described [34] and pixel density of individual spots was analysed on inverted images with Image-J software. A heatmap was generated using GraphPad Prism 7.

### Procarta multiplex assay

Protein extracted as above from individual samples was tested for presence of CCL2, CCL3, CCL4, CCL5, CCL11, CXCL1, CXCL8, CXCL10 and CXCL12 by ProcartaPlex assay (Affymetrix eBioscience, Hatfield, UK). The assay was performed using 10μg protein per sample according to manufacturer's guidelines and detected on a Bio-Rad Bio-Plex 200 HTF multiplex assay system. A sample was considered valid for analysis is the CXCL8 value was higher than100 pg/200 μg protein.

Of the 1051 samples analysed using ProcartaPlex, exclusions were made due to three failed experimental runs (error in experimental standards), CXCL8 value (<100 pg/200 μg protein) or missing clinical data rendering an analysable sample set of 648. When stratified by HPV status (to include only hrHPV positive samples) our final sample set (*N* = 476) incorporated 181 samples with normal cytology/histology, 58 CIN1, 100 CIN2, 135 CIN3, 1 Cervical Glandular intraepithelial neoplasia (CGIN) and 1 early invasive squamous cancer (Table 2).

**Table 2:** Stratification of sample set by cytology/histology classification and HPV status

Cytology/ Histology results	hrHPV negative	hrHPV positive	Total
**Normal^1^**	120 (39.9%)	181 (60.1%)	301 (48.2%)
**CIN1**	19 (24.7%)	58 (75.3%)	77 (12.3%)
**CIN2**	6 (5.7%)	100 (94.3%)	106 (17.0%)
**CIN3**	4 (2.9%)	135 (97.1%)	139 (22,2%)
**Cervical glandular intraepithelial neoplasia**	0 (0.0%)	1 (100%)	1 (0.2%)
**Early invasive squamous cancer**	0 (0.0%)	1 (100%)	1 (0.2%)
**Total**	149	476	625

^1^Normal as defined by cytology or no biopsy required at colposcopy or histologically confirmed normal.

### Statistical analysis

Procartaplex results were visualised by boxplots to compare the distribution of chemokine values for CIN2+ or CIN3+ and the Wilcoxon–Mann–Whitney test used to compare the distributions chemokine values at each disease threshold.

To assess the ability to detect disease, initially each chemokine alone (univariate analysis) was evaluated in its ability to make predictions for both disease thresholds using logistic regression. We then considered the possibility of significant interactions between chemokines and conducted tests of interaction between all pairs of chemokines. This resulted in 15 tests of interaction for each disease threshold. To account for the multiplicity of testing for each threshold, a Bonferonni correction was applied to the significance level used and interactions with *p*-values less than α = 0.05/15 = 0.003 were considered to be statistically significant and investigated further. All chemokines and significant interactions were included in a multivariable logistic regression model. Model reduction was performed using backwards selection to ascertain if a statistically optimal model could be found, with chemokines removed using the least significant chemokine first according to the likelihood ratio test. The model reduction stopped when all remaining chemokines were significant at the 5% level. In addition, models with a combination of chemokines deemed to be of clinical interest were also considered.

A model build, with both univariate and multivariate estimation, was conducted using the training set, which was a randomly chosen sample of 50% of the data. The ability of the model to predict diseases status was then evaluated using the remaining 50% of the data. The overall predictability of the model was assessed using the area under the curve (AUC) and the sensitivity and specificity of the model was assessed using the probability of disease threshold which optimises the AUC. In the interests of comparability, the same statistical models were compared for both end points. All statistical analysis was performed using the Statistics package R version 3.2.3.
